# Propofol-related biological alterations and incidence of propofol infusion syndrome in status epilepticus: a 10-year cohort study

**DOI:** 10.3389/fneur.2025.1753979

**Published:** 2026-01-21

**Authors:** Chanez Djahel, Clémence Marois, Léa Cosme, Zineb Hayatou, Nesrine Braham Ghedira, Quentin Calonge, Vincent Navarro, Adam Celier, Dominique Bonnefont-Rousselot, Randa Bittar, Sophie Demeret, Aurélie Hanin

**Affiliations:** 1 AP-HP.Sorbonne Université, Pitié-Salpêtrière-Charles Foix University Hospital, Department of Metabolic Biochemistry, DMU BioGeMH, Paris, France; 2 AP-HP.Sorbonne Université, Hôpital de la Pitié-Salpêtrière, Neuro-Intensive Care Unit, Paris, France; 3 AP-HP, Hôpital de la Pitié-Salpêtrière, Sorbonne Université, Epilepsy Unit and Department of Clinical Neurophysiology, ERN-Epicare, Paris, France; 4 Sorbonne Université, Institut du Cerveau - Paris Brain Institute - ICM, Inserm, CNRS, AP-HP, Hôpital de la Pitié Salpêtrière, Paris, France; 5 Université Paris Cité, UTCBS, CNRS, INSERM, Paris, France; 6 Université Paris Cité, Faculté de Pharmacie, Paris, France

**Keywords:** biological biomarkers, intensive care unit, propofol, propofol infusion-related syndrome, status epilepticus

## Abstract

**Introduction:**

Continuous anesthetics are often required for the management of refractory status epilepticus. While essential to achieving seizure control, these agents carry significant adverse effects. In particular, propofol may induce hypotension, respiratory depression, metabolic acidosis, or pancreatitis, with the most feared complication being propofol infusion syndrome (PRIS).

**Methods:**

The primary objective was to investigate the biological effects of propofol in patients with status epilepticus admitted to the neurointensive care unit of Pitié-Salpêtrière Hospital (Paris, France) for at least 48 h between September 2015 and October 2024. Twenty biological parameters reflecting acid–base balance, organ function, lipid metabolism, and inflammation were analyzed. To capture delayed effects, biological tests from day *t + 1* were used to assess propofol exposure on day *t*. A linear mixed-effects model was applied, with each marker as the dependent variable, propofol exposure (including patients who did not receive propofol as the reference group) or dose of propofol as the fixed-effect predictor, and patient as a random effect. A secondary objective was to determine the incidence of PRIS in this tertiary referral center for the management of refractory and super-refractory status epilepticus.

**Results:**

A total of 235 patients were enrolled, of whom 51% received propofol for at least 1 day. We collected biological data over 2,407 patient-days, including 1,086 (45%) under propofol infusion. Propofol use was associated with lipid dysregulation, characterized by increased triglycerides, decreased LDL-cholesterol and HDL-cholesterol, along with impaired renal function, and mild acute respiratory acidosis, reflected by elevated pCO_2_, and reduced pH and phosphate levels. Propofol exposure was also associated with a pro-inflammatory profile, characterized by elevated CRP, procalcitonin, leukocyte counts, and an altered neutrophil-to-lymphocyte ratio, independent of the patient’s clinical inflammatory status. Over the past 10 years, only four cases of likely or most likely PRIS cases were identified.

**Discussion:**

Prolonged propofol infusions warrant routine monitoring of specific biological markers. Our study identifies key parameters to follow and confirms that PRIS remains rare in specialized centers where patients are closely monitored and risk factors are carefully considered.

## 1 Introduction

Status epilepticus (SE) is a common neurological emergency, defined by the persistence or recurrence of epileptic seizures, and associated with significant morbidity and mortality (1–4). In up to 30% of cases, seizures persist or recur despite treatment with two appropriate intravenous antiseizure medications, including a benzodiazepine, a condition known as refractory SE (5). Management of refractory SE often requires admission to the intensive care unit (ICU) and the use of continuous intravenous anesthetic drugs (CIVADs) (6, 7).

Early administration of CIVADs has been associated with improved SE control and lower mortality (8, 9). Similarly, coma induction after the first-line treatment was associated with shorter SE duration and ICU and hospital stays (10). Several CIVADs can be used in SE, including propofol, midazolam, barbiturates, or ketamine (11). A multicenter retrospective study found that midazolam and propofol have comparable efficacy and safety, with a more favorable benefit–risk profile than barbiturates (12, 13). Propofol is sometimes preferred over midazolam in the management of SE in adults because of its pharmacokinetics, notably a shorter half-life, which may facilitate earlier weaning (5). However, prolonged infusions are not without adverse effects and may induce respiratory-tract infections, ventilator dependence, difficult weaning, hemodynamic instability, increased mortality, and poor neurological outcomes (14).

Several biological complications have been associated with propofol administration, including hypertriglyceridemia, respiratory acidosis, and organ dysfunction. These abnormalities are thought to reflect the lipophilic nature of the treatment and early metabolic stress induced by propofol. At the most severe end of this spectrum lies propofol infusion syndrome (PRIS), a rare but potentially lethal complication, reported after prolonged and/or high-dose infusions (5, 15–21). Patients with PRIS typically present with multiple organ system failures, including impaired cardiovascular contractility, metabolic and lactic acidosis, rhabdomyolysis, lipidaemia, acute renal failure, and hepatomegaly (22–24). It seems more likely to occur in pediatric patients (25), or in the presence of additional risk factors such as carbohydrate depletion, severe illness, concomitant administration of catecholamines, glucocorticoids, or ketogenic diet (22, 26), or in patients with a history of mitochondrial disorders or beta-oxidation defects (27, 28). An analysis of the FDA Event Reporting System (FAERS) database also identified epilepsy as a significant risk factor for both PRIS development and mortality (29). Consequently, clinicians have suggested avoiding initiation of the ketogenic diet within 24 h of starting propofol infusions to prevent potentially fatal PRIS (30). Similarly, an etiological workup for mitochondrial dysfunctions should be performed when a patient is admitted for SE and develops PRIS (31).

In Europe, regulatory authorities have recommended close monitoring for metabolic acidosis, rhabdomyolysis, elevated creatine kinase levels, and heart failure, with dose reduction or discontinuation of propofol if these conditions occur (24, 32). However, detailed information on the effect of propofol dose and exposure on biological parameter levels remains limited, and clinicians currently lack reliable biological warning signs to anticipate propofol-related toxicity and inform timely therapeutic decisions in the management of refractory SE.

In this study, our first objective was to investigate the detailed effect of propofol exposure and dose on 20 biological parameters reflecting acid–base balance, organ function, lipid metabolism, and inflammation in patients with SE. Our second objective was to determine the incidence of PRIS over a 10-year period in a tertiary referral center specializing in the management of refractory and super-refractory SE.

## 2 Materials and methods

### 2.1 Study design, settings, and participants

We conducted a retrospective, single-center cohort study of patients admitted for the management of SE to the Neurointensive Care Unit at the Pitié-Salpêtrière Hospital (Paris, France), a national tertiary referral center for refractory cases.

We included patients aged at least 15 years old, admitted with an International Statistical Classification of Diseases and Related Health Problems, 10th Revision (ICD-10) hospital discharge code G41 for SE between September 2015 and October 2024, who remained in the ICU for more than 48 h and had biological biomarkers measured at least twice during their stay. All medical records were reviewed, and patients who did not meet the International League Against Epilepsy definition for SE or the ACNS electrophysiological criteria on continuous EEG monitoring (3, 33), or whose SE had resolved and for whom CIVADs had been weaned off upon admission, were excluded.

This study was conducted in accordance with the Strengthening the Reporting of Observational Studies in Epidemiology (STROBE) guidelines (34). This study was conducted according to French legislation and authorized by the French data protection authority CNIL (No. 2211991). According to the Declaration of Helsinki, patients or relatives were informed that their anonymized data would be used in this study.

### 2.2 Data collection

Clinical and biological data were extracted directly from medical records.

For all patients, we collected the following information: sex, age at admission, refractoriness of the SE [i.e., refractory SE, super-refractory SE, prolonged super-refractory SE (35)], etiology group [i.e., acute, remote, progressive, unknown (33)], personal history of epilepsy, number of CIVADs used to manage SE, duration of SE (the end of SE was defined as either the cessation of CIVADs without recurrence of seizures or the end of seizures in patients who did not require anesthesia), and duration of the ICU stay.

For each day of the ICU stay, we collected data on the use of propofol, the maximum daily propofol dose, ketogenic diet administration, noradrenaline infusion, and intravenous methylprednisolone. In patients who received propofol for at least 1 day, we also extracted information on preexisting factors associated with, or reported in, PRIS or mitochondrial disease, such as consanguinity, migraines, visual or auditory impairments, cardiac failure, ataxia, cerebellar syndrome, diabetes, or thyroid disorders (15, 36). For patients who had been transferred to our unit from another hospital, the timing of CIVADs introduction and the propofol doses administered before admission were not collected.

We also assessed the inflammatory status for each day with a blood draw in the ICU. Patients were noted in an inflammatory state if they presented with fever (≥38 °C), were prescribed antibiotics, or if daily medical records mentioned an inflammatory condition (e.g., septic shock, pneumonia, etc.).

### 2.3 Biological measurements

In patients who received propofol, we extracted the biological values daily from admission, continued throughout the entire duration of propofol infusion, and then for up to 7 days after the propofol wean. In patients who did not receive propofol, the biological values were collected daily during the first 7 days after admission, when available. In case of multiple daily measurements for a biomarker, the first value was retained. Blood samples were usually collected in the early morning, but sampling times could vary across parameters.

We extracted daily values for 20 biological parameters selected to reflect acid–base balance (pH, pCO_2_, pO_2_, bicarbonate, lactate, phosphate), organ function (high-sensitivity troponin T, creatine kinase, aspartate aminotransferase [AST], alanine aminotransferase [ALT], creatinine), lipid metabolism (total cholesterol, triglycerides, high-density lipoprotein cholesterol [HDL-cholesterol], low-density lipoprotein cholesterol [LDL-cholesterol]), and inflammation (C-reactive protein [CRP], procalcitonin, leukocyte count, and neutrophil and lymphocyte percentages). All biological parameters were measured on lithium heparin tubes, except lipid-related biomarkers, which were measured in serum gel tubes.

All biochemical assays were performed on the automated Cobas® platform (Roche Diagnostics, Basel, Switzerland). Complete blood counts and leukocyte differential counts were obtained by flow cytometry using a Sysmex XN® hematology analyzer (Sysmex Corporation, Kobe, Japan). Arterial blood gases (pH, pCO_2_, pO_2_, bicarbonate, and lactate) were measured by direct potentiometry with a blood gas analyzer (ABL 825®, Radiometer, Copenhagen, Denmark), and phosphate levels were determined by indirect potentiometry. High-sensitivity cardiac troponin T and procalcitonin were assessed by electrochemiluminescence immunoassay, and CRP by immunoturbidimetry. Liver enzymes (AST and ALT) and creatine kinase were quantified using kinetic enzymatic assays, whereas creatinine was measured with an enzymatic method traceable to the isotope dilution mass spectrometry reference procedure. Lipid profile included total cholesterol, triglycerides, and HDL-cholesterol, all analyzed by enzymatic methods; LDL-cholesterol was calculated using the Friedewald formula, or directly measured by colorimetric enzymatic assay when triglycerides exceeded 3.4 g/L.

### 2.4 PRIS likelihood

PRIS likelihood was determined by expert review based on the presence of characteristic clinical and biological features reported in the literature (including metabolic acidosis or hyperlactatemia, rhabdomyolysis, hypertriglyceridemia, cardiac dysfunction or arrhythmias, and multiorgan failure), their temporal relationship with propofol exposure, and improvement after propofol discontinuation, while taking into account propofol dose and duration, concomitant treatments such as ketogenic diet, and the absence of a more plausible alternative diagnosis. Following these criteria, cases were classified as ruled out, unlikely, likely, or most likely for PRIS.

### 2.5 Statistical analysis

Statistical analyses were conducted using R Studio software (version 2025.09.0). A two-tailed *p* < 0.05 was considered statistically significant.

Descriptive statistics were reported as counts and percentages for categorical variables, and as median and interquartile range (IQR) for continuous variables. Comparisons between groups were performed using the chi-square test for categorical variables. Continuous variables were compared using the Wilcoxon test.

For each biological parameter, we examined whether its daily value was influenced by propofol exposure. Linear mixed-effects models with a random intercept for each patient were used to account for repeated measurements during the ICU stay. The main fixed effect was propofol administration on that day, or the maximum daily propofol dose, and each biomarker serves as the dependent variable. Two analyses were performed: (1) the association between propofol administration at day *t* and biological values measured on the same day (*t*), and (2) the association between propofol administration at day *t* and biological values measured on the following day (*t + 1*). For each parameter, we reported the estimated effect of propofol, its 95% confidence interval (CI), and the *p*-value (Supplementary Figure 1).

Higher doses of propofol may have been administered to control seizure burden in patients with concurrent sepsis. Therefore, to account for inflammation, we conducted two complementary models. The first provided the adjusted effect of propofol, correcting for daily inflammatory status, as defined above. The second included a “propofol x inflammation” interaction term to test whether the effect of propofol differed depending on the inflammation. When the interaction was significant after Benjamini-Hochberg correction, we reported the stratum-specific slopes (propofol effects on days with and without inflammation).

## 3 Results

### 3.1 Study cohort

Between September 2015 and October 2024, 303 patients with an ICD-10 code of G41 were hospitalized in the neurointensive care unit for more than 48 h. After reviewing each medical record, 235 patients met the inclusion criteria. Most patients presented with refractory SE (82%, 193 patients), with acute etiology being the most frequent group (44%). Propofol was administered to 121 (51%) patients.

Patients had a median age of 53 years (interquartile range [IQR] 35–66), and 61% were male. A total of 108 patients (46%) had a prior history of epilepsy. Patients received a median of 1 [IQR 0–2] CIVADs during their ICU stay. The patients admitted with refractory SE and who did not require CIVADs presented with focal refractory SE or generalized refractory SE for which general anesthesia had to be avoided (e.g., elderly or comorbid patients for whom initiating mechanical ventilation would, for example, be considered unreasonable), and that was controlled by adding a third anti-seizure medication. Detailed clinical and demographic characteristics are summarized in Table 1.

**Table 1:** Clinical and demographic information of the cohort.

Number of patients	235
Number of hospitalizations stays	252
Number of male (%)	143 (61%)
Age, years, median [IQR]	53 [35–66]
Number of patients who received propofol at least one day, number (%)	121 (51%)
Previous history of epilepsy, number (%)	108 (46%)
SE refractoriness	-
Refractory status epilepticus, number (%)	193 (82%)
Super-refractory status epilepticus, number (%)	76 (32%)
Prolonged super-refractory status epilepticus, number (%)	40 (17%)
Etiology groups	-
Acute, number (%)	103 (44%)
Remote, number (%)	87 (37%)
Progressive, number (%)	38 (16%)
Unknown, number (%)	7 (3%)
Number of continuous anesthetics used, median [IQR]	1 [0–2]
Duration of the SE, days, median [IQR]	4 [1–12]
Duration of the ICU stay, days, median [IQR]	11 [5–25]

ICU, intensive care unit; IQR, interquartile range; RSE, refractory status epilepticus; SE, status epilepticus.

Patients who received propofol had a longer SE duration (10 [IQR 4–23] vs. 1 [IQR 0–4] days, *p* < 0.001) and longer ICU stays (16 [IQR 9–37] vs. 6 [IQR 4–15] days, *p* < 0.001) than those who did not receive it. They also received catecholamines more frequently (46% vs. 14%; *p* < 0.001). As expected, there was a higher proportion of refractory, super-refractory, or prolonged super-refractory SE in patients who received propofol as the third or fourth line therapy, compared to those who did not receive it. There were no differences in age, sex, prior history of epilepsy, or SE etiology between groups (Supplementary Table 1).

### 3.2 Effect of propofol infusion on biological biomarkers

Biological parameter levels were collected over 2,407 patient-days, with a median of 7 days per patient (IQR 4–11, maximum 81). Of these, 1,086 measurements (45%) were obtained under propofol infusion, while 1,321 (55%) were obtained without propofol.

**Figure 1:**
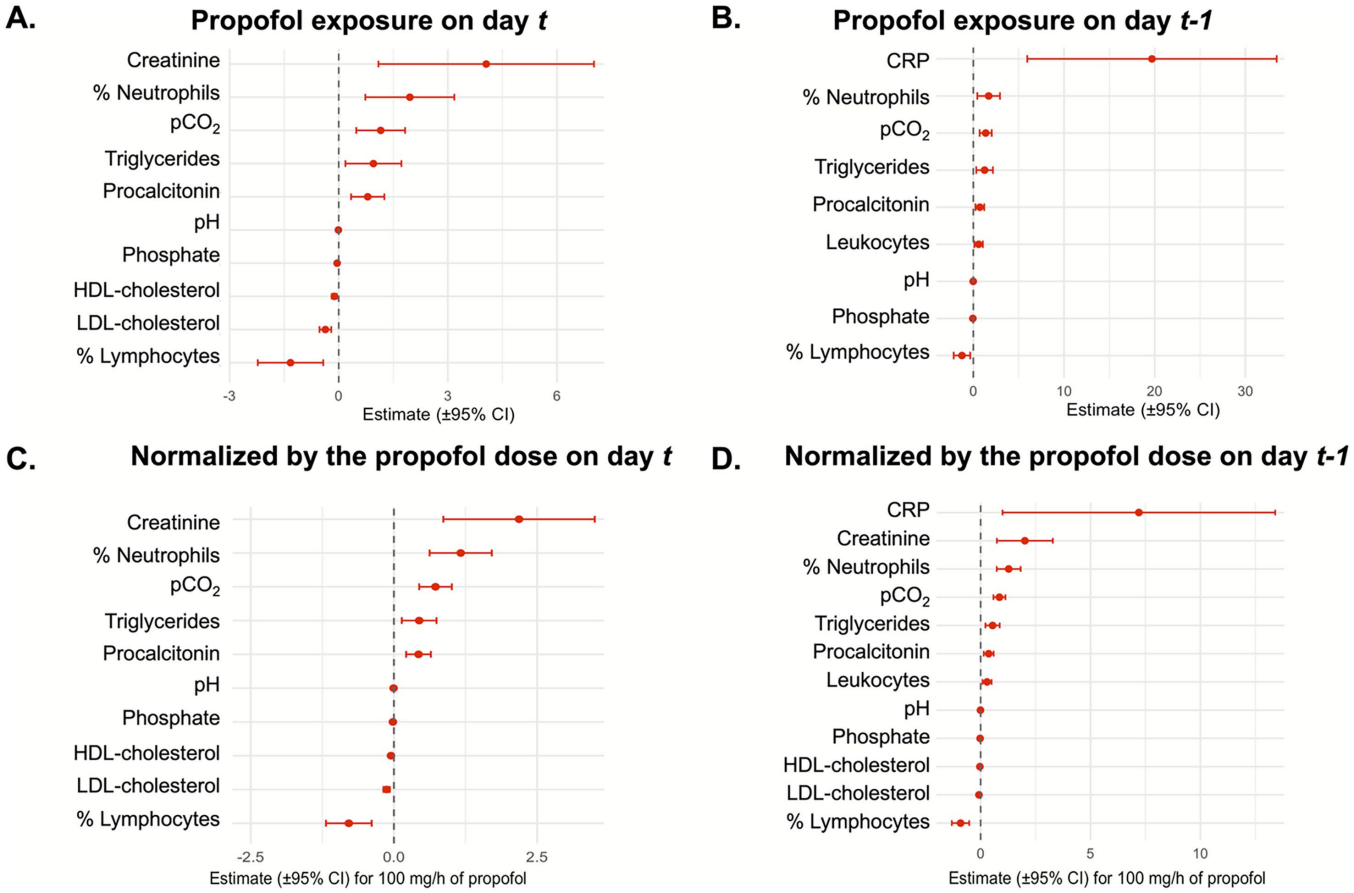
Forest plots representing the estimated effects of propofol exposure or dose of propofol on biological markers. The forest plots represent the effect of propofol exposure on the day of sample collection **(A)** or the following day **(B)**; or of the maximal dose of propofol on the day of sample collection **(C)** or the following day **(D)**.

Propofol exposure was associated with significant changes in 10 biological parameters (Supplementary Table 2; Figure 1A). Increases were observed in pCO_2_ (+1.15 mmHg [95% CI 0.48–1.83]), creatinine (+4.06 μmol/L [95% CI 1.09–7.02]), triglycerides (+0.95 g/L [95% CI 0.18–0.72]), neutrophil percentage (+1.96% [95% CI 0.73–3.18]), and procalcitonin (+0.80 μg/L [95% CI 0.34–1.25]). Decreases were noted in phosphate (−0.044 mmol/L [95% CI −0.077 – −0.012]), pH (−0.01 [95% CI −0.018 – −0.0029]), LDL-cholesterol (−0.37 g/L [95% CI −0.53 – −0.21]), HDL-cholesterol (−0.12 g/L [95% CI −0.19 – −0.05]), and lymphocyte percentage (−1.33% [95% CI −2.23 – −0.43]).

To account for the delayed effects of propofol on biological parameters, additional analyses were performed to assess the impact of propofol exposure on day *t* on parameters measured on day *t + 1*. A total of 2,158 patient-days were included in this analysis, comprising 1,069 (50%) with propofol and 1,089 (50%) without propofol on day *t*. Significant differences were found in nine parameters (Table 2; Figure 1B). As on the day of sample collection, propofol exposure on the preceding day was associated with significant increases in pCO_2_ (+1.36 mmHg [95% CI 0.70–2.03]), triglycerides (+1.24 g/L [95% CI 0.32–2.16]), neutrophil percentage (+1.69% [95% CI 0.44–2.94]), and procalcitonin (+0.72 μg/L [95% CI 0.24–1.20]). Additional increases in CRP (+20.0 mg/L [95% CI 5.9–33.5]) and leukocyte count (+0.58 G/L [95% CI 0.12–1.05]) further supported the pro-inflammatory effect of propofol exposure. Decreases in pH (−0.014 [95% CI −0.020 – −0.0073]), phosphate (−0.05 mmol/L [95% CI −0.08 – −0.018]), and lymphocyte percentage (−1.25% [95% CI −2.17 – −0.34]) were also confirmed. In contrast, creatinine, HDL-cholesterol, and LDL-cholesterol were no longer significantly different.

**Table 2:** Effects of propofol exposure on day t on the biological levels measured on the following day (t + 1).

Biological biomarkers	With propofol (*n* = 1,069)	Without propofol (*n* = 1,089)	Estimate [95% CI]	*p*-value
pH	7.43 [7.40–7.47]	7.44 [7.41–7.48]	−0.014 [−0.020; −0.0073]	<0.001
pCO_2_, mmHg	39.6 [35.6–43]	37.5 [33.6–40.9]	1.36 [0.70; 2.03]	<0.001
pO_2_, mmHg	86.0 [76.0–105.0]	88.0 [75.0–108.0]	−1.07 [−5.19; 3.04]	0.61
Bicarbonate, mmol/L	25.8 [23.2–28.8]	25.2 [22.4–28.1]	0.08 [−0.32; 0.48]	0.70
Lactate, mmol/L	1.0 [0.6–1.4]	1.1 [0.8–1.5]	−0.057 [−0.15; 0.035]	0.23
Phosphate, mmol/L	0.99 [0.79–1.2]	1.0 [0.81–1.2]	−0.05 [−0.08; −0.018]	0.0028
Troponin, ng/L	34.7 [12.1–77.4]	66.7 [23.2–152.0]	−4.81 [−103.9; 94.3]	0.92
Creatine kinase, UI/L	56.0 [26.0–211.5]	159.0 [54.0–536.2]	−3101.9 [−8995.9; 2792.1]	0.30
AST, UI/L	38.0 [23.0–70.8]	36.0 [22.0–70.5]	5.0 [−11.8; 21.8]	0.56
ALT, UI/L	44.0 [25.0–99.3]	38.0 [19.0–101.3]	2.6 [−7.2; 12.4]	0.60
Creatinine, μmol/L	46.0 [37.0–61.0]	53.0 [42.0–71.0]	2.79 [−0.20; 5.78]	0.068
Total cholesterol, g/L	1.72 [1.34–2.17]	1.61 [1.21–1.80]	0.059 [−0.21; 0.33]	0.67
Triglycerides, g/L	2.12 [1.50–3.58]	1.23 [0.79–1.84]	1.24 [0.32; 2.16]	0.0085
HDL-cholesterol, g/L	0.29 [0.19–0.34]	0.36 [0.31–0.48]	−0.073 [−0.15; 0.00013]	0.060
LDL-cholesterol, g/L	0.76 [0.43–1.19]	0.85 [0.73–1.09]	−0.14 [−0.31; 0.021]	0.11
CRP, mg/L	63.3 [22.9–128.8]	49.3 [17.9–117.3]	20.0 [5.9; 33.5]	0.0053
Procalcitonin, μg/L	0.19 [0.078–0.57]	0.17 [0.08–0.44]	0.72 [0.24; 1.20]	0.0037
Leukocyte, G/L	9.68 [7.0–13.2]	9.48 [7.11–12.6]	0.58 [0.12; 1.05]	0.015
Neutrophil (%)	74.9 [65.1–82.5]	73.2 [63.6–79.8]	1.69 [0.44; 2.94]	0.0082
Lymphocyte (%)	12.6 [8.0–20.5]	15.0 [10.1–23.0]	−1.25 [−2.17; −0.34]	0.0073

Values are represented as median [interquartile range]. The *p*-value was obtained by a linear mixed-effects regression analysis. ALT, alanine aminotransferase; AST, aspartate aminotransferase; CRP, C-reactive protein; HDL, high-density lipoprotein; LDL, low-density lipoprotein.

### 3.3 Effect of the dose of propofol on biological biomarkers

We next evaluated the effect of the maximal daily dose of propofol on biological parameters.

On the day of sample collection, each 100 mg/h increase in propofol infusion was associated with significant changes in the same 10 biological parameters identified for propofol exposure (Supplementary Table 3; Figure 1C). Increases were noted in pCO_2_ (+0.73 mmHg [95% CI 0.44–1.01]), creatinine (+2.18 μmol/L [95% CI 0.86–3.51]), triglycerides (+0.44 g/L [95% CI 0.14–0.74]), procalcitonin (+0.43 μg/L [95% CI 0.21–0.65]), and neutrophil percentage (+1.17% [95% CI 0.62–1.71]). Conversely, decreases were observed in phosphate (−0.018 mmol/L [95% CI −0.032 – −0.0037]), pH (−0.0059 [95% CI −0.0091 – −0.0028]), LDL-cholesterol (−0.13 g/L [95% CI −0.18 – −0.071]), HDL-cholesterol (−0.051 g/L [95% CI −0.075 – −0.028]), and lymphocyte percentage (−0.79% [95% CI −1.19 – −0.39]).

When examining the delayed effect of propofol dose on day *t* on biological parameters measured on day *t + 1*, significant differences were again observed for multiple markers (Table 3; Figure 1D). As on the day of sample collection, increases were observed in pCO_2_ (+0.86 mmHg [95% CI 0.58–1.13]), triglycerides (+0.54 g/L [95% CI 0.22–0.87]), creatinine (+2.01 μmol/L [95% CI 0.74–3.29]), neutrophil percentage (+1.28% [95% CI 0.73–1.82]), and procalcitonin (+0.37 μg/L [95% CI 0.14–0.59]). Additional increases in CRP (+7.20 mg/L [95% CI 1.0–13.4]) and leukocyte count (+0.29 G/L [95% CI 0.086–0.50]) supported the pro-inflammatory effect of propofol, as previously observed with overall exposure. Decreases were confirmed for pH (−0.0078 [95% CI −0.010 – −0.0051]), phosphate (−0.024 mmol/L [95% CI −0.039 – −0.0094]), lymphocyte percentage (−0.92% [95% CI −1.31 – −0.52]), HDL-cholesterol (−0.036 g/L [95% CI −0.061 – −0.010]), and LDL-cholesterol (−0.077 g/L [95% CI −0.13 – −0.022]).

**Table 3:** Effects of an increase of 100 mg/h of propofol on day t on biological levels measured on day t + 1.

Biological biomarkers	Estimate [95% CI]	*p*-value
pH	−0.0078 [−0.010; −0.0051]	<0.001
pCO_2_, mmHg	0.86 [0.58; 1.13]	<0.001
pO_2_, mmHg	−0.56 [−2.28; 1.16]	0.52
Bicarbonate, mmol/L	0.085 [−0.081; 0.25]	0.32
Lactate, mmol/L	0.0067 [−0.032; 0.045]	0.73
Phosphate, mmol/L	−0.024 [−0.039. -0.0094]	0.0013
Troponin, ng/L	−6.59 [−41.1; 27.9]	0.71
Creatine kinase, UI/L	−1030.0 [−3368.3; 1308.3]	0.39
AST, UI/L	1.60 [−5.53; 8.74]	0.66
ALT, UI/L	2.55 [−1.62; 6.73]	0.23
Creatinine, μmol/L	2.01 [0.74; 3.29]	0.0020
Total cholesterol, g/L	−0.0083 [−0.12; 0.099]	0.88
Triglycerides, g/L	0.54 [0.22; 0.87]	0.0012
HDL-cholesterol, g/L	−0.036 [−0.061; −0.010]	0.011
LDL-cholesterol, g/L	−0.077 [−0.13; −0.022]	0.018
CRP, mg/L	7.20 [1.0; 13.4]	0.024
Procalcitonin, μg/L	0.37 [0.14; 0.59]	0.0014
Leukocyte, G/L	0.29 [0.086; 0.50]	0.0055
Neutrophil (%)	1.28 [0.73; 1.82]	<0.001
Lymphocyte (%)	−0.92 [−1.31; −0.52]	<0.001

The *p-*value was obtained by a linear mixed-effects regression analysis. ALT, alanine aminotransferase; AST, aspartate aminotransferase; CRP, C-reactive protein; HDL, high-density lipoprotein; LDL, low-density lipoprotein.

### 3.4 Influence of the inflammatory condition on the propofol effect

**Figure 2:**
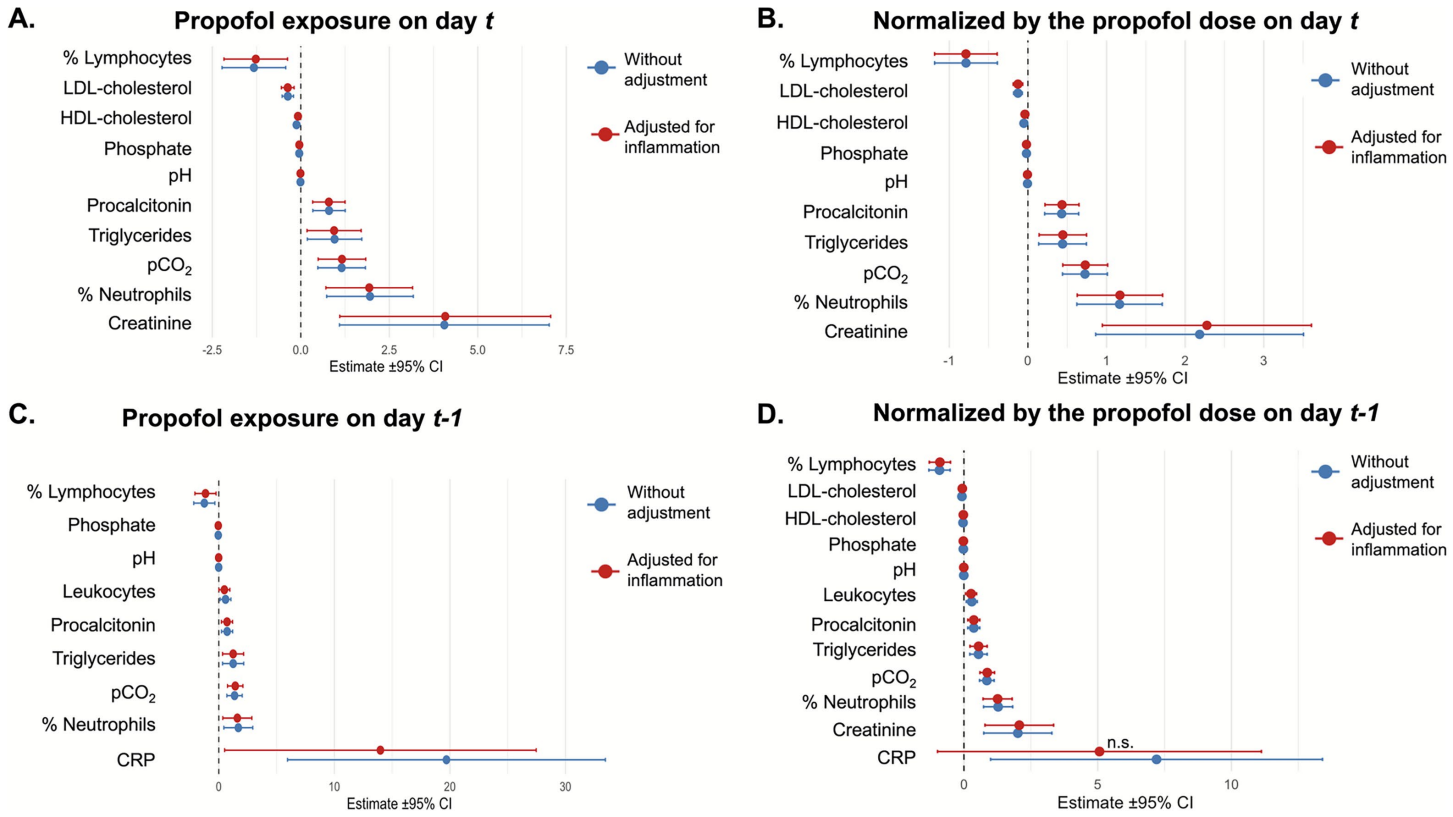
Forest plots representing the effect of propofol adjusted for the patient’s daily inflammatory status. The forest plots represent the effect of propofol exposure adjusted for the patient’s daily inflammatory status on the day of sample collection **(A)** or the following day **(C)**; or the maximal dose of propofol on the day of sample collection **(B)** or the following day **(D)**.

Adjusting for daily inflammatory status did not modify the set of biological parameters showing significant changes, except for CRP, which was no longer significant when assessing the effect of propofol dose on day *t* on CRP measured on day *t + 1* (Figure 2).

We next investigated whether the presence of inflammation (i.e., the propofol-inflammation interaction) modified the strength or direction of the propofol effect. The magnitude of the propofol effect on the day of sample collection was reduced in the presence of inflammation for both the neutrophil percentage (+0.65% [CI 95% −0.77 – 2.07] vs. +4.94% [CI 95% 2.83–7.04], *p* = 0.012), and the lymphocyte percentage (−0.48% [−1.52–0.57] vs. −3.15% [−4.70 – −1.61], *p* = 0.023), with similar findings observed when considering propofol dose (Table 4). The effect of propofol on bicarbonate levels also differed according to the inflammatory status, with a significant increase observed in the presence of inflammation (Table 4). Similarly, an increase in pCO2 was observed when higher doses of propofol were administered during inflammation (Table 4). No significant differences were observed when considering propofol exposure on the preceding day.

**Table 4:** Effects of the interaction propofol-inflammation on the day of sample collection.

Biological biomarkers	*p*-value for the propofol exposure	Slope with inflammation	Slope without inflammation	*p*-value for the dose of propofol	Slope with inflammation for 100 mg/h of propofol	Slope without inflammation for 100 mg/h of propofol
pH	0.72	-	-	0.63	-	-
pCO_2_, mmHg	0.14	-	-	0.0032	1.02 [0.70; 1.35]	0.019 [−0.47; 0.51]
pO_2_, mmHg	0.17	-	-	0.053	-	-
Bicarbonate, mmol/L	0.023	0.45 [0.018; 0.89]	−0.69 [−1.35; −0.040]	<0.001	0.30 [0.11; 0.48]	−0.36 [−0.64; −0.089]
Lactate, mmol/L	0.31	-	-	0.23	-	-
Phosphate, mmol/L	0.91	-	-	0.64	-	-
Troponin, ng/L	0.34	-	-	0.53	-	-
Creatine kinase, UI/L	0.72	-	-	0.64	-	-
AST, UI/L	0.94	-	-	0.85	-	-
ALT, UI/L	0.72	-	-	0.60	-	-
Creatinine, μmol/L	0.88	-	-	0.60	-	-
Total cholesterol, g/L	0.94	-	-	0.93	-	-
Triglycerides, g/L	0.88	-	-	0.37	-	-
HDL-cholesterol, g/L	0.86	-	-	0.85	-	-
LDL-cholesterol, g/L	0.72	-	-	0.66	-	-
CRP, mg/L	0.72	-	-	0.53	-	-
Procalcitonin, μg/L	0.72	-	-	0.76	-	-
Leukocyte, G/L	0.64	-	-	0.23		
Neutrophil (%)	0.012	0.65 [−0.77; 2.07]	4.94 [2.83; 7.04]	0.0032	0.62 [0; 1.24]	2.64 [1.67; 3.61]
Lymphocyte (%)	0.023	−0.48 [−1.52; 0.57]	−3.15 [−4.70; −1.61]	0.011	−0.44 [−0.90; 0.011]	−1.71 [−2.41; −1.0]

*p-*values were adjusted with Benjamini-Hochberg procedures. The slopes for the inflammatory and non-inflammatory conditions were reported when the p-value was significant. ALT, alanine aminotransferase; AST, aspartate aminotransferase; CRP, C-reactive protein; HDL, high-density lipoprotein; LDL, low-density lipoprotein.

### 3.5 Cases of propofol infusion-related syndromes

In our cohort, 22 patients received propofol at doses exceeding 4 mg/kg/h for more than 48 h (37), and PRIS was suspected in four of them, mostly based on biological abnormalities. A review of medical records for all other patients exposed to propofol identified five additional cases in whom PRIS was suspected, including two patients for whom propofol had already been discontinued upon admission to our ICU (Table 5). One patient had a known mitochondrial encephalomyopathy with lactic acidosis and stroke-like episodes (MELAS) associated with hypoacusis. None of the other patients had a history of consanguinity, migraines, ataxia, cerebellar syndrome, diabetes, or thyroid disorders. Six of the nine patients received concomitant catecholamines (patients #1, 4, 6, 7, 8, and 9), four were on a ketogenic diet (patients #2, 6, 7, and 8), and three (patients #7, 8, and 9) received corticosteroids. No patient died because of PRIS. Table 5 summarizes their clinical and demographic characteristics. The nine cases were reviewed in detail by experts (CM, AC, SD) and classified as *ruled out*, *unlikely*, *likely*, or *most likely* for PRIS. Only four cases were considered *likely* or *most likely* (Table 5). Among them, one was under a ketogenic diet and three received catecholamines.

**Table 5:** Clinical and demographic information of patients experiencing probable or confirmed propofol infusion syndrome.

Patient	Age	Sex	Personal medical history	SE etiology	Maximal propofol dose (mg/h)	Symptoms suggesting PRIS	Ketogenic diet	Management for the PRIS	Opinion of experts on the PRIS status	GOS-E at ICU discharge
#1	19	M	None	Cryptogenic NORSE	240	Hyperlactatemia Hypertriglyceridemia Liver and renal acute failure Elevated creatine kinase Hemodynamic failure	No	Propofol stopped	Likely	3
#2	18	M	None	Cryptogenic NORSE	Not reported (propofol stopped before admission)	Hypertriglyceridemia	Yes	Propofol stopped	Not enough information provided to conclude about the PRIS	3
#3	35	M	None	Subdural hematoma	400	Hepatic cytolysis Cholestasis Elevated creatine kinase and fibrinogen	No	Propofol stopped	Ruled out. No definite criteria for PRIS	4
#4	66	F	Cerebral vasculitis Hypertension Depression	Neuro COVID	250	Hypertriglyceridemia	No	Propofol stopped	Ruled out. Only hypertriglyceridemia. (Propofol was given for COVID management)	1
#5	56	M	MELAS	MELAS	Not reported (propofol stopped before admission)	Hyperlactatemia Hypertriglyceridemia Elevated creatine kinase, troponin, and AST/ALT	No	Propofol stopped Extrarenal clearance	Most likely	3
#6	27	M	Epilepsy	Myoclonic-astatic epilepsy	400	Hypertriglyceridemia Rhabdomyolysis	Yes	Ketogenic diet stopped	Unlikely. Mild rhabdomyolysis (creatine kinase up to 560 UI/L) and hypertriglyceridemia	3
#7	32	F	None	Cryptogenic NORSE	440	Hypertriglyceridemia	Yes	Propofol reduced Plasmapheresis	Unlikely. Only hypertriglyceridemia	3
#8	26	M	None	Cryptogenic NORSE	300	Acidosis Hypertriglyceridemia Hyperlactatemia	Yes	Propofol and ketogenic diet stopped	Likely	3
#9	34	F	Pericarditis	Cryptogenic NORSE	400	Cardiac arrythmias Elevated creatine kinase, triglycerides	No	VA-ECMO Propofol stopped	Most likely	5

F, female; GOS-E, Glasgow Outcome Extended Score; M, male; MELAS, mitochondrial encephalomyopathy with lactic acidosis and stroke-like episodes; NORSE, new-onset refractory status epilepticus; VA-ECMO, veno-arterial extracorporeal membrane oxygenation.

## 4 Discussion

In this study, we demonstrated that propofol exposure in patients with SE induces significant biological alterations, including marked dyslipidemia (increased triglycerides, decreased HDL- and LDL-cholesterol), mild respiratory acidosis, transient renal dysfunction, and signs of innate immune activation. These effects were generally modest and reversible, but clinically relevant, as they may represent early markers of metabolic stress under prolonged sedation. Despite the severity of our population, which comprises a large proportion of refractory SE cases, only 3% (4/121) of patients who received propofol developed a *likely* or *most likely* PRIS, suggesting that with appropriate monitoring, this rare but potentially lethal complication remains uncommon.

Propofol exposure led to marked dyslipidemia characterized by increased triglycerides and decreased HDL- and LDL-cholesterol levels, with clear dose-dependent relationships. These findings are consistent with the lipophilic nature of propofol and previous reports showing hypertriglyceridemia in up to 45% of patients who received propofol infusions (38–40). Although some triglyceride elevations were mild in our cohort, increases are known risk factors for pancreatitis (41), and were reported to be associated with 30-day mortality in intensive care patients (42). Thus, systematic monitoring of triglyceride levels during long-term or high-dose propofol infusions is warranted.

We also confirmed that propofol can induce mild respiratory acidosis, evidenced by lower pH and phosphate and higher pCO_2_ levels. Propofol is known to disturb mitochondrial energy metabolism by inhibiting key enzymes of the electron transport chain and disrupting fatty acid *β*-oxidation through blockage of carnitine palmitoyltransferase (CPT-I), thereby reducing the entry of long-chain fatty acids into mitochondria (43–45). This inhibition limits ATP production and can lead to an energy deficit and accumulation of lactate. In our study, no significant changes were observed in lactate or bicarbonate levels in the presence of propofol, suggesting that the effects of propofol exposure were modest and primarily reflected hypoventilation rather than systemic metabolic disturbance (46). However, under inflammatory conditions and with higher propofol doses, a greater increase in pCO_2_ and a compensatory rise in bicarbonate were observed, supporting the hypothesis that inflammation may exacerbate propofol’s impact on respiratory and metabolic homeostasis. Because these changes were not observed on the following day, they likely represent transient, direct effects of the drug.

Evidence of organ dysfunction was limited. Transient increases in creatinine were observed on the day of propofol exposure, without persistent effect, consistent with mild and reversible renal dysfunction. No significant changes were found in hepatic (AST, ALT) or muscular (creatine kinase, troponin) markers, suggesting that propofol-induced organ toxicity was limited in our cohort. Interestingly, although median creatinine levels were slightly lower under propofol in unadjusted analyses, mixed-effects modeling revealed a modest positive association, illustrating a Simpson’s paradox driven by differences in disease severity and repeated within-patient measurements (47).

Propofol exposure was also associated with signs of innate immune activation, including elevated procalcitonin, increased neutrophil-to-lymphocyte ratio, and, on the following day, higher CRP and leukocyte counts. While dyslipidemia is a well-recognized effect of propofol, inflammatory activation has received less attention in the literature. This pro-inflammatory signature may reflect cytokine induction or immune cell activation secondary to metabolic stress. However, previous reports suggested that propofol has inhibiting effects on immunity (48, 49). We wondered if these findings could be biased by the use of higher doses of propofol in patients with septic conditions who are at risk of seizure recurrence (50). When we accounted for patients’ inflammatory status, the propofol’s effect remained, suggesting that the observed changes were not solely due to the inflammatory status of the patient. However, the magnitude of propofol’s effects decreased, particularly for CRP on day *t + 1*, suggesting that CRP may reflect the underlying inflammatory context rather than a direct pharmacologic effect of propofol.

In our cohort, only four patients who received propofol developed a *likely* or *most likely* PRIS. The incidence of PRIS in our cohort is similar to what was previously described in patients who received propofol for at least 24 h (51). Consistent with historical PRIS descriptions (15, 22), all four cases exhibited hypertriglyceridemia, and three out of the four patients developed rhabdomyolysis. Renal and heart failure were often described in PRIS patients, while autopsy may reveal hepatic microvesicular steatosis (52–55). In our cohort, only one patient had cardiac failure, which required VA-ECMO. PRIS cases classically result from a combination of mitochondrial dysfunction, impaired fatty acid oxidation, and catecholamine-driven metabolic stress, with the ketogenic diet and mitochondrial disorders being known as risk factors. Our findings align with this spectrum, with elevated lactate present in three PRIS cases, supporting the role of mitochondrial injury.

Although many observed abnormalities were modest in the overall cohort, they remain clinically important because they may precede more severe mitochondrial dysfunction and help clinicians recognize early toxicity. Clearer monitoring strategies, particularly of triglycerides, acid–base status, renal function, and inflammatory markers, could represent early metabolic perturbations, help guide dose adjustments, and inform decisions to continue, wean off, or switch sedation during refractory SE. While these changes do not in themselves constitute PRIS, they highlight the need for vigilance, as clinicians currently lack objective biomarkers to detect early toxicity. Future work should aim to define biomarker thresholds to guide safe long-term propofol use.

This study provides an overview of biological marker disturbances associated with propofol exposure in a large cohort of patients with SE. However, several limitations should be acknowledged. First, biological samples were collected only once daily, most often in the morning, whereas propofol infusion rates varied throughout the day. Consequently, same-day associations may underestimate peak drug effects, and the delayed analyses (day *t + 1*) likely provide a more reliable reflection of the biological impact of propofol. However, most patients received propofol over several days. As a result, when evaluating the effect of exposure on day *t-1* on biological markers at day *t*, most patients were still receiving propofol at day *t*. This introduces an overlap in exposure and limits our ability to isolate the specific impact of the previous day’s treatment. Additionally, although prolonged propofol exposure is known to increase the risk of biological adverse effects, we were unable to assess the effect of cumulative dose on biomarker levels because information on CIVAD administration and dosing before admission at our center was not available. Similarly, severity-of-illness scores (e.g., APACHE II, SOFA) were not available because most patients were transferred to our unit after propofol initiation in referring centers and therefore could not be included as confounders in the analysis. The information regarding the SE type (convulsive vs. non-convulsive; focal vs. generalized) has not been collected. Although most patients with refractory and super-refractory status epilepticus underwent continuous EEG monitoring, this study did not examine the effects of propofol on the electroencephalographic patterns, nor stratify patients based on their EEG findings. In our neurointensive care unit, propofol is titrated in mg/h rather than mg/kg/h, which may influence dose–response interpretation across patients of different body weights. To minimize bias related to inflammation, we analyzed the effect of each patient’s daily inflammatory status; however, daily clinical notes with information regarding inflammation were not available for all patients, particularly the oldest cases, and antibiotic use did not always correspond to an inflammatory state. Moreover, metabolic disturbances observed in our cohort may also be influenced by other confounding factors, including multiorgan failure, renal replacement therapy, and concomitant treatments (e.g., midazolam, ketamine, catecholamines, or ketogenic diet). Future studies should specifically investigate the contribution of these factors. As with inflammation, it is possible that clinicians used higher sedation doses in response to greater patient severity, including metabolic disturbances; therefore, we cannot fully rule out the possibility of reverse causality in our findings. Additionally, because only a few cases of PRIS were identified, comparative analyses of patients with or without PRIS were not possible. Finally, the observational design limits causal inference, and residual confounding by indication cannot be excluded, as patients receiving propofol generally had more severe or prolonged SE than those who did not receive propofol, which may itself contribute to the observed biological abnormalities.

## 5 Conclusion

In summary, propofol exposure in SE patients induces transient, dose-dependent alterations in lipid metabolism, respiratory acid–base balance, renal function, and inflammatory markers, while clinically significant PRIS remains rare under close monitoring. These findings support the need for systematic biological surveillance, particularly of triglycerides, acid–base parameters, and inflammatory markers, during prolonged or high-dose propofol infusion in critically ill patients.

## Data Availability

The raw data supporting the conclusions of this article will be made available by the authors, without undue reservation.
