# Supplementary material for: Propofol-related biological alterations and incidence of propofol infusion syndrome in status epilepticus: a 10-year cohort study
Source: Front Neurol. 2026 Jan 21;16:1753979. doi: 10.3389/fneur.2025.1753979 (PMC12867868; doi:10.3389/fneur.2025.1753979)
Supplement: Supplementary file 1 [file Table_1.docx]

**Supplementary Material**

**Supplementary Table 1: Comparisons of clinical data between patients who received propofol or not.**

|  | Hospitalizations with propofol used (n=127) | Hospitalizations without propofol used (n=125) | P-value |
| --- | --- | --- | --- |
| Age, years, median [IQR] | 51 [32-63] | 55 [39-67] | 0.083 |
| Number of male (%) | 78 (61%) | 76 (61%) | >0.99 |
| Previous history of epilepsy, number (%) | 59 (46%) | 66 (53%) | 0.38 |
| SE refractoriness | - | - | - |
| Refractory status epilepticus, number (%) | 119 (94%) | 87 (70%) | <0.001 |
| Super-refractory status epilepticus, number (%) | 72 (57%) | 8 (6%) | <0.001 |
| Prolonged super-refractory status epilepticus, number (%) | 39 (31%) | 3 (2%) | <0.001 |
| Etiology groups | - | - | - |
| Acute, number (%) | 62 (49%) | 41 (33%) | 0.063 |
| Remote, number (%) | 43 (34%) | 57 (46%) |  |
| Progressive, number (%) | 18 (14%) | 24 (19%) |  |
| Unknown, number (%) | 4 (3%) | 3 (2%) |  |
| Number of continuous anesthetics used, median [IQR] | 2 [1-3] | 0 [0-1] | <0.001 |
| Duration of the SE, days, median [IQR] | 10 [4-23] | 1 [0-4] | <0.001 |
| Duration of the ICU stay, days, median [IQR] | 16 [9-37] | 6 [4-15] | <0.001 |

Descriptive statistics were reported as counts and percentages for categorical variables, and as median and interquartile range (IQR) for continuous variables. Comparisons between groups were performed using the chi-square test for categorical variables. Continuous variables were compared using the Wilcoxon test.

Abbreviations: ICU, intensive care unit; IQR, interquartile range; SE, status epilepticus

**Supplementary Table 2: Effects of propofol exposure on biological levels on the day of sample collection**

|  | With propofol  (n=1086) | Without propofol  (n=1321) | Estimate [95% CI] | P-value |
| --- | --- | --- | --- | --- |
| pH | 7.44 [7.39-7.47] | 7.44 [7.40-7.47] | -0.01 [-0.018; -0.0029] | 0.0063 |
| pCO_2_, mmHg | 39.4 [35.4-43.1] | 37.7 [34.0-41.4] | 1.15 [0.48; 1.83] | <0.001 |
| pO_2_, mmHg | 88.0 [76.0-108.0] | 89.0 [76.0-112.0] | 2.44 [-2.13; 7.01] | 0.30 |
| Bicarbonate, mmol/L | 25.7 [22.9-28.7] | 25.1 [22.3-27.9] | 0.12 [-0.26; 0.50] | 0.54 |
| Lactate, mmol/L | 1.0 [0.6-1.5] | 1.1 [0.8-1.5] | -0.018 [-0.13; 0.097] | 0.76 |
| Phosphate, mmol/L | 0.99 [0.78-1.22] | 1.01 [0.82-1.19] | -0.044 [-0.077; -0.012] | 0.0068 |
| Troponin, ng/L | 34.7 [12.8-82.2] | 46.4 [16.6-116] | 1.93 [-74.1; 78.0] | 0.96 |
| Creatine kinase, UI/L | 68.5 [28.8-236.3] | 157.0 [55.0-485.8] | -2195.7 [-6455.8; 2064.4] | 0.31 |
| AST, UI/L | 38 [23-66] | 35 [22-67] | 1.56 [-13.9; 17.0] | 0.84 |
| ALT, UI/L | 42 [24-88] | 36 [19-90] | 1.01 [-8.60; 10.6] | 0.84 |
| Creatinine, µmol/L | 47 [37-62] | 54 [42-72] | 4.06 [1.09; 7.02] | 0.0074 |
| Total cholesterol, g/L | 1.71 [1.30-2.12] | 1.61 [1.29-1.81] | 0.0045 [-0.24; 0.25] | 0.97 |
| Triglycerides, g/L | 2.08 [1.41-3.53] | 1.25 [0.87-1.92] | 0.95 [0.18; 1.72] | 0.016 |
| HDL-cholesterol, g/L | 0.29 [0.19-0.34] | 0.42 [0.33-0.51] | -0.12 [-0.19; -0.05] | 0.0013 |
| LDL-cholesterol, g/L | 0.76 [0.43-1.19] | 0.85 [0.74-1.11] | -0.37 [-0.53; -0.21] | 0.0012 |
| CRP, mg/L | 52.8 [14.0-121.7] | 39.0 [10.8-95.8] | 11.3 [-1.28; 24.0] | 0.079 |
| Procalcitonin, µg/L | 0.16 [0.07-0.54] | 0.16 [0.07-0.51] | 0.80 [0.34; 1.25] | <0.001 |
| Leukocyte, G/L | 9.65 [6.92-13.3] | 9.65 [7.21-12.7] | 0.28 [-0.17; 0.73] | 0.22 |
| Neutrophil (%) | 75.2 [65.4-83] | 73.6 [63.6-80.7] | 1.96 [0.73; 3.18] | 0.0018 |
| Lymphocyte (%) | 12.4 [7.9-20.4] | 14.9 [9.7-22.7] | -1.33 [-2.23; -0.43] | 0.0039 |

Values are represented as median [interquartile range].

The p-value was obtained by a linear mixed-effects regression analysis.

Abbreviations: ALT, alanine aminotransferase; AST, aspartate aminotransferase; CRP, C-reactive protein; HDL, high-density lipoprotein; LDL, low-density lipoprotein

**Supplementary Table 3: Effects of an increase of 100 mg/h of propofol on biological levels on the day of sample collection**

|  | Estimate [95% CI] | P-value |
| --- | --- | --- |
| pH | -0.0059 [-0.0091; -0.0028] | <0.001 |
| pCO_2_, mmHg | 0.73 [0.44; 1.01] | <0.001 |
| pO_2_, mmHg | 1.61 [-0.36; 3.57] | 0.11 |
| Bicarbonate, mmol/L | 0.10 [-0.058; 0.26] | 0.21 |
| Lactate, mmol/L | 0.0075 [-0.042; 0.057] | 0.77 |
| Phosphate, mmol/L | -0.018 [-0.032; -0.0037] | 0.014 |
| Troponin, ng/L | -4.67 [-35.3; 26.0] | 0.77 |
| Creatine kinase, UI/L | -788.2 [-2604.1; 1027.8] | 0.40 |
| AST, UI/L | -1.58 [-8.26; 5.09] | 0.64 |
| ALT, UI/L | 0.079 [-4.08; 4.24] | 0.97 |
| Creatinine, µmol/L | 2.18 [0.86; 3.51] | 0.0012 |
| Total cholesterol, g/L | -0.018 [-0.12; 0.085] | 0.74 |
| Triglycerides, g/L | 0.44 [0.14; 0.74] | 0.0047 |
| HDL-cholesterol, g/L | -0.051 [-0.075; -0.028] | <0.001 |
| LDL-cholesterol, g/L | -0.13 [-0.18; -0.071] | 0.0014 |
| CRP, mg/L | 4.68 [-1.02; 10.4] | 0.11 |
| Procalcitonin, µg/L | 0.43 [0.21; 0.65] | <0.001 |
| Leukocyte, G/L | 0.17 [-0.034; 0.37] | 0.10 |
| Neutrophil (%) | 1.17 [0.62; 1.71] | <0.001 |
| Lymphocyte (%) | -0.79 [-1.19; -0.39] | <0.001 |

The p-value was obtained by a linear mixed-effects regression analysis.

Abbreviations: ALT, alanine aminotransferase; AST, aspartate aminotransferase; CRP, C-reactive protein; HDL, high-density lipoprotein; LDL, low-density lipoprotein
