# Supplementary material for: Propofol-related biological alterations and incidence of propofol infusion syndrome in status epilepticus: a 10-year cohort study
Source: Front Neurol. 2026 Jan 21;16:1753979. doi: 10.3389/fneur.2025.1753979 (PMC12867868; doi:10.3389/fneur.2025.1753979)
Supplement: Supplementary file 2 [file Image_1.pdf]

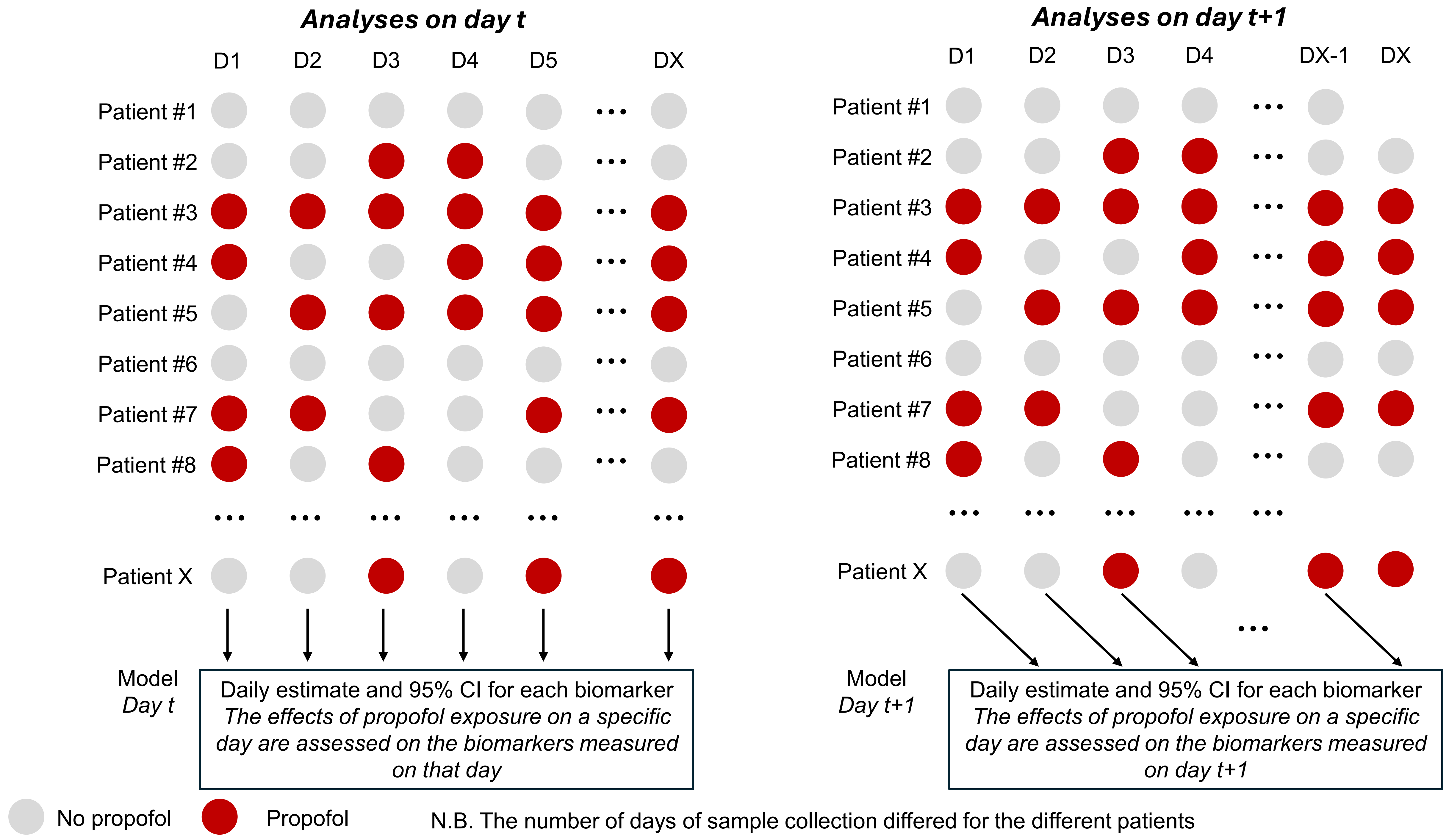
Analyses on day t
D1
D2
D3
D4
D5
DX
Patient #1
Patient #2
Patient #3
Patient #4
Patient #5
Patient #6
Patient #7
Patient #8
Patient X
Model
Day t
Daily estimate and 95% CI for each biomarker
The effects of propofol exposure on a specific day are assessed on the biomarkers measured on that day
Analyses on day t+1
D1
D2
D3
D4
DX-1
DX
Patient #1
Patient #2
Patient #3
Patient #4
Patient #5
Patient #6
Patient #7
Patient #8
Patient X
Model
Day t+1
Daily estimate and 95% CI for each biomarker
The effects of propofol exposure on a specific day are assessed on the biomarkers measured on day t+1
No propofol
Propofol
N.B. The number of days of sample collection differed for the different patients
